# Toward Resilient Wireless Sensor Networks: A Virtualized Perspective

**DOI:** 10.3390/s20143902

**Published:** 2020-07-13

**Authors:** Adnan Rashid, Tommaso Pecorella, Francesco Chiti

**Affiliations:** Department of Information Engineering, Università di Firenze, 50139 Firenze, Italy; adnan.rashid@unifi.it (A.R.); tommaso.pecorella@unifi.it (T.P.)

**Keywords:** IoT, resilient networks, RPL, 6LoWPAN, IEEE 802.15.4, NFV, security, single point of failure

## Abstract

The Internet of Things (IoT) has been one of the main focus areas of the research community in recent years, the requirements of which help network administrators to design and ensure the functionalities and resources of each device. Generally, two types of devices—constrained and unconstrained devices—are typical in the IoT environment. Devices with limited resources—for example, sensors and actuators—are known as constrained devices. Unconstrained devices includes gateways or border routers. Such devices are challenging in terms of their deployment because of their connectivity, channel selection, multiple interfaces, local and global address assignment, address resolution, remote access, mobility, routing, border router scope and security. To deal with these services, the availability of the IoT system ensures that the desired network services are available even in the presence of denial-of-service attacks, and the use of the system has become a difficult but mandatory task for network designers. To this end, we present a novel design for wireless sensor networks (WSNs) to address these challenges by shifting mandatory functionalities from unreliable to reliable and stable domains. The main contribution of our work consists in addressing the core network requirements for IoT systems and pointing out several guidelines for the design of standard virtualized protocols and functions. In addition, we propose a novel architecture which improves IoT systems, lending them more resilience and robustness, together with highlighting and some important open research topics.

## 1. Introduction

The system of either directly or indirectly interrelated computing devices, including mechanical, electrical, electronics or digital machines and animals or people, connected to human daily life is known as the Internet of Things (IoT) or Internet of Everything (IoE). IoT has the unique feature of generating and transferring data over the Internet without requiring human-to-human or human-to-computer interaction, enabling distributed, resilient and autonomous systems. The application of IoT in society spans different areas, such as commercial (medical and healthcare, transportation, V2X communications, building and home automation), military (battlefield and ocean of things), infrastructure (metropolitan scale deployments, energy management and environmental monitoring), consumer (smart home and handicapped), and industrial applications (agriculture and manufacturing).

IoT is primarily driven by low-cost constrained devices, in which the main constraints are in the memory and computational capacity available to each device. Moreover, IoT devices are usually meant to be run on a battery or by energy-harvesting: thus, low energy consumption is the main goal. In order to keep the energetic cost to the minimum, IoT devices use specialized communication protocols, called low power and lossy networks (LLNs).

**Figure 1:**
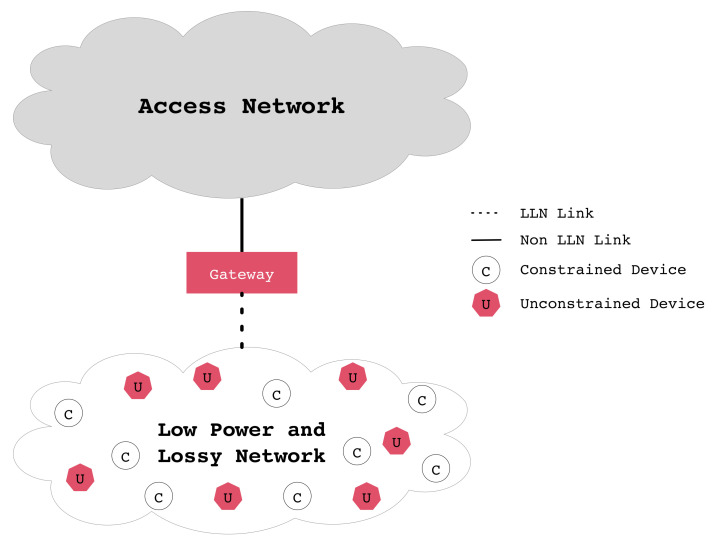
Abstract view of elements of interest in the Internet of Things (IoT) paradigm.

Figure 1 shows a logical representation of a generic wireless sensor network (WSN). A gateway device is required at the edge of the WSN domain to provide Internet connectivity to all of its attached constrained and unconstrained devices. Unconstrained devices usually have more capabilities and access to more reliable power sources.

As with conventional networks, the essential security requirements (confidentiality, authenticity, integrity, accountability and availability) are also mandatory for WSNs [1]. In particular, they might be different depending on the application scenario; however, they are always important. A security breach can lead to severe consequences, ranging from the loss of users’ personal data to the safety of whoever relies on the WSN’s capabilities. The last point is especially important in applications such as Smart Cities or Industrial IoT (IIoT). Securing devices and their communications in a distributed way on a large scale, preserving information security on the collected data and controlling information are very challenging and important issues that cannot be neglected.

In this paper, we analyze the core requirements of IoT systems, while outlining the major critical points which have not yet been addressed by the standards and proposing an architecture to mitigate the most severe risks that are actually affecting IoT networks. The proposed architecture generally corresponds to the others already discussed in standards, but there are many different features that we highlight that can improve the resilience of the IoT systems. The following are the main contributions of this work.

- We move the core functionalities of protocols—i.e., IEEE 802.15.4 [2], 6LoWPAN Border Router (6LBR) [3,4] and the Virtual DoDAG root [5]—from an uncertain network to a more stable and controllable network.
- We achieve the coordination of egress points (EPs) (i.e., gateways/edge routers), which perform the functionality of the IPv6 Backbone Router (6BBR) [6] as well as the DoDAG root.
- We use the Virtual DoDAG for the Fog Layer, which helps in the coordination among multiple DoDAG roots.
- We use the Fog Layer to increase scalability and help constrained devices achieve time-critical applications.
- We synchronize multiple EPs; furthermore, we ensure network services and claim that our architecture is more resilient.

The rest of the paper is organized as follows: in the next section, we explain different scenarios and their requirements in terms of their availability, scalability, security and management. In Section 3, applicable standards for the IoTs with the integration of classical standards are discussed. The proposed architecture is finally presented in Section 5. Finally, we highlight several open issues and directions for future work in Section 6.

## 2. Service Requirements

IoT networks are an enabling technology for the so-called Fourth Industrial Revolution (Industry 4.0), Smart Cities, Smart Health, etc. It is envisioned that IoT systems will be based on heterogeneous network types, along with Cloud and Fog-based systems [1]. As a consequence, we can expect a wide range of technologies; e.g., Fifth Generation (5G), Bluetooth (802.15.1), LiFi (802.15.7r1), WiFi (802.11), WPANs (802.15.4), Ethernet, etc. Despite the wide variety of systems, IoT systems will almost always share the same general concept shown in Figure 1, where a number of unreliable devices are connected to the Internet, providing a globally reliable service by means of redundancy, cooperative functions, etc. As an example, multihop systems are based on the idea that, even if a node fails, other paths can be quickly established.

In the present paper, we are interested in the overall system reliability; i.e., the determination of the actual problems that can affect the service as a whole. Without loss of generality, we can list the following requirements that are common to any IoT system.

### 2.1. Availability

**Figure 2:**
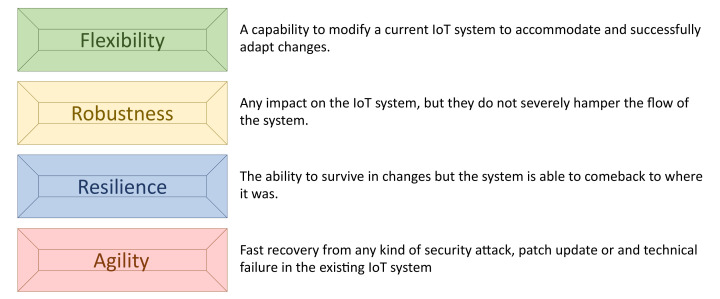
Main capabilities used to improve availability.

As with conventional networks, the essential security requirements—i.e., confidentiality, authenticity, integrity, accountability and availability—are also mandatory for IoT systems. Availability ensures that the desired network services are available even in the presence of denial-of-service (DoS) attacks. As mentioned in Figure 2, it is important for network administrators and designers to consider this requirement by ensuring flexibility, robustness, resilience and agility capabilities.

### 2.2. Scalability

This requirement describes the ability to scale the entire network by ensuring stability and competitiveness when demand is increased. According to [7], one network size hardly fits for all scenarios, since requirements are typically time-variant. However, one size of the network cannot solve all future market demands. It may become difficult and challenging to ensure the same services with the same quality of service (QoS) after scaling the existing network. Ensuring the scalability requirement in IoT systems also become very challenging when dealing with heterogeneous devices, because the heterogeneity of devices encompasses different applications, protocols, mobility, hardware etc.

### 2.3. Security

As in conventional networks, the five security functions—i.e., confidentiality, authentication, accountability, availability and integrity—are also mandatory for IoT systems [1]. We discuss availability independently, because it becomes very challenging in single point of failure (SPoF) scenarios that we highlighted in Section 1. IoT is certainly the most complex and still open area of network security because embedded devices are concerned with vulnerabilities and there is no good way to patch them. The chip manufacturers adopt its firmware and software to optimize their incentives. Device chips are selected by the vendors based on features and price, and the vendors perform certain manipulations if anything is required by the chip software and firmware; thus, only device functionality is their main focus. On the other hand, end users are usually not able or allowed to patch the system, or if they can, then they have limited information about when and how to patch. As a result, hundreds of millions of Internet-connected devices in the IoT are vulnerable to attacks [8]. Sensors have definitely become vulnerable, because they allow attackers to inject malicious code and data into the network. This is also a threat for actuators, where the attacker can manipulate the operation of machinery and other devices in the IoT system.

### 2.4. Management

This is a process of administrating and controlling devices and their functionalities in the entire IoT ecosystem. Generally, there are two categories of management: first is network management, where the network administrator performs analysis and maintains the performance of entire IoT system, and the second is network security, where security-related process are managed.

#### 2.4.1. Network Management

Network management is widely considered one of the hard problems of networking and continues to be the focus of much research even in IoT systems. The management of hundreds of millions of heterogeneous, small objects becomes very challenging due to their heterogeneous nature. This includes performance management, fault analysis, location management, mobility management, the provisioning of QoS and any kind of service management.

#### 2.4.2. Network Security Management

Securing and controlling heterogeneous IoT devices is very challenging, because IoT security operation and management includes routine IoT security evaluation, security logs and the automatic identification of the security events based on the best practice polices. The security management platform (centralized or distributed) can be provided for policy configuration, policy orchestration and policy execution. Nevertheless, it is essential to ensure homogeneous security polices for the entire IoT system.

## 3. Relevant Standards for IoT

In this section, we discuss the standard technologies that are relevant for our scenario. As stated previously, we can partition the network from a topological point of view into two main parts:

- The wireless segment, in which devices use unreliable communication systems and are mostly resource-constrained, and
- The wired segment, where we can assume we are able to use high-speed, reliable, and secure networking technologies.

Referring to Figure 1, gateway devices reside between the two parts (wired and wireless). Without loss of generality, we can assume that the wired section is reliable and secure, thanks to the use of the Ethernet, software-defined networks (SDN), network virtualization, etc. In contrast, we will assume that the wireless segment is a multihop IoT system, enabled by any multihop technology; e.g., Bluetooth Mesh, IEEE 802.15.4, etc. For the present discussion, however, the exact physical and MAC standard is not relevant, as our focus is on upper layers.

**Figure 3:**
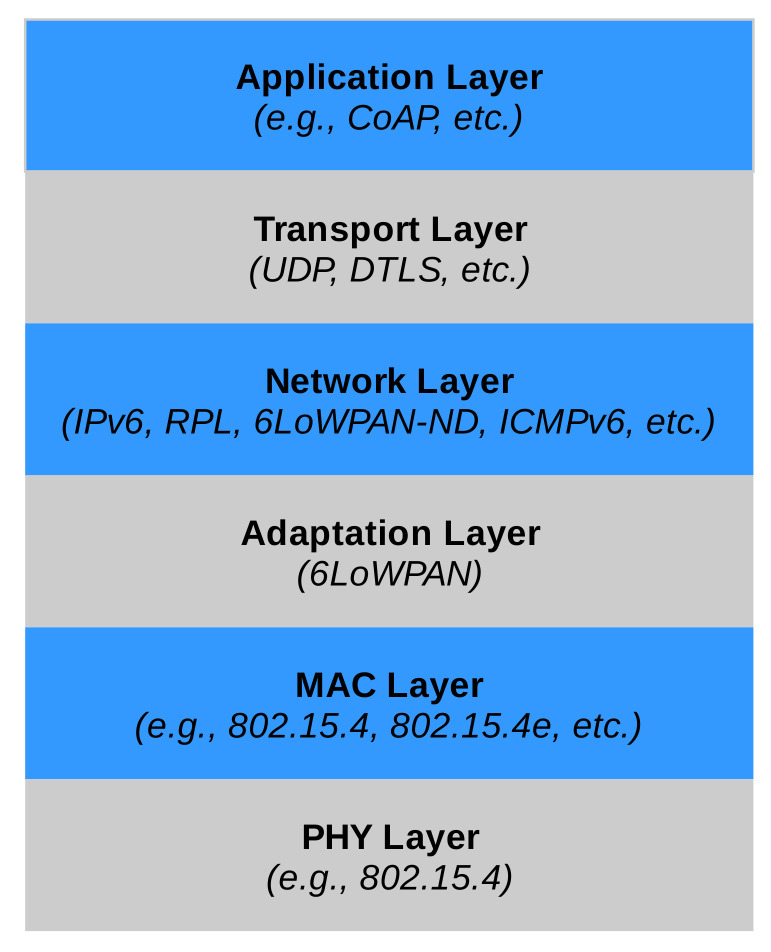
IoT-oriented IP protocol stack.

In the following, we will assume that the IoT system is based on IP standards; in particular, IPv6. As shown in Figure 3, the IP protocol stack for IoT systems is largely identical to the “normal” IP stack, with some notable exceptions that can affect the system resilience.

The use of IPv6 for resource-constrained networks and devices can lead to some inefficiencies. For this reason, a set of “adaptation” protocols have been defined by the 6Lo IETF Working Group. In particular, 6LoWPAN [9,10] allows the compression of the IPv6 header, while 6LoWPAN-ND [3] mainly helps in optimizing Neighbor Discovery. 6LoWPAN is (almost) a stateless compression system, and it does not cause particular issues; in contrast, 6LoWPAN-ND can lead to network failures, as we will show below. Another point worth analyzing is the routing protocol, as it can be a potential source of issues.

### 3.1. Neighbor Discovery Optimization

Traditional IPv6 Neighbor Discovery (IPv6-ND) [11] is used for router discovery, address resolution, DAD (duplicate address detection) and redirecting messages, along with prefix and parameter discovery. 6LoWPAN-Neighbor Discovery (6LoWPAN-ND) optimized this protocol for the Low-Power and Lossy Networks (LLNs) [3]. In an LLN, devices are classified according to their role: 6LoWPAN Node (6LN), 6LoWPAN Router (6LR), and 6LoWPAN Border Router (6LBR). A 6LN is a “normal” device, while 6LR refers to devices which are able to relay messages and 6LBR is a device responsible for managing the network. A very important difference between IPv6-ND and 6LoWPAN-ND is that IPv6-ND is completely distributed, while 6LoWPAN-ND is a centralized protocol: all the IPv6-ND functionalities normally using multicast messaged are substituted by a request–reply mechanism between 6LNs and the 6LBR, with 6LRs acting as relays.

The introduction of a request–reply system helps the LLN, as multicast messages are not efficient in LLN and they might be not supported at all in some architectures. On the other hand, a centralized entity is a potential security and reliability problem.

It is worth noting that 6LoWPAN-ND is also responsible for network prefix dissemination, DAD and IP address registration, and the network parameters must be periodically refreshed. As a consequence, each 6LN must have a stable connection with the 6LBR. An erratic connection or an incorrect configuration of the 6LoWPAN-ND timers can lead to network failures.

### 3.2. Network Routing

Routing is usually the critical point in a resilient network. As a matter of fact, the Internet is also “resilient” thanks to its decentralized routing approach. However, the SDN paradigm has recently emerged as a viable alternative to “classical” routing approaches. SDN enables, among other things, better and finer-grained traffic engineering, faster network reconfiguration, per-flow routing, etc.

No matter which approach is used—SDN or IP routing—the implications for network resiliency are evident: a failure in a forwarding device might jeopardize the network. For this reason, it is worth recalling the basic ideas behind both approaches.

- **IP-based routing:** IP routing heavily depends on the type of network being considered. In the wired part, there are well-known protocols, such as RIP, OSPF, EIGRP, etc., whose resilience has been extensively studied. As a matter of fact, the resilience is in this case almost entirely dependent on the routing protocol convergence time.

Wireless multihop networks require special routing protocols, as every node (not only the routers) actually participate in the forwarding scheme. Although many routing schemes has been proposed for multihop and ad-hoc networks, IPv6 Routing Protocol for Low-Power and Lossy Networks (RPL) [5] is a routing protocol built to fulfill the specific IoT scenarios. As a consequence, we will focus on RPL in the following discussion.
In RPL, the network topology is oriented toward a sink (or, in RPL terms, a root node). All the paths are built to originate from the sink node, and its existence is central for the whole network (RPL enables also other kind of paths, but for the sake of brevity, we will not consider them in the present discussion). Although RPL builds redundant network paths to prevent node and link failures, and although the recovery time from network disruption is limited, the root node is a potential issue. It is possible to have multiple different RPL roots in an IoT network, but this is not usually a good solution, as it increases the network complexity and the memory requirements in the nodes.

- **SDN forwarding:** The SDN approach decouples the routing function from the forwarding function. In an SDN network, all the forwarding nodes only keep a forwarding table, which is built by a centralized controller. The controller creates the forwarding tables by having a complete topological knowledge of the network and possibly other data such as the switches’ queue occupancy, link resource utilization, etc.

The SDN approach was initially proposed for wired networks, and it is now a well-known technology used in many scenarios. Due to the benefits offered by SDN, there have been several recent proposals to extend it to wireless links [12]. Nevertheless, one central requirement for SDN is to have a secure, reliable and fast link between any switching device and the SDN controller. This limitation makes its applicability in wide multi-hop networks quite problematic, if not entirely impossible.

## 4. Approaches to Resilience in IoT

As discussed in Section 2, network resilience depends on four elements: availability, scalability, security and management. In order to fulfill these requirements, each network element should be resilient to a variety of attacks, and the network itself should be self-healing. As noted previously, a single IoT device can be attacked; however, a powerful network design should prevent a case in which a single device failure can disrupt the network as a whole. As a consequence, we believe that the very first requirement is to avoid or mitigate the presence of an SPoF in the network.

The most recent research contributions toward network resilience in IoT networks are summarized in Table 1. We considered here only works focusing on protocols above layer 2, and we will assume, without loss of generality, that an unmodified IEEE 802.15.4 standard [2] is being used. This assumption is justified by the fact that using a “custom” L2 protocol usually leads to increased device costs and long-term device resupply issues.

From Table 1, we can see that the SPoF issue is still an open problem. On the other hand, fulfilling the network requirements that we addressed in Section 2 is ongoing research because it depends on dynamic changes in the market. These requirements are directly proportional to the market demands [1]. In the literature review, our selection criteria spanned SPoF, core network requirements and different emerging technologies such as SDN, NFV (network function virtualization) and the Fog/Cloud.

The architectures presented in [14,16,17,18,22,25,37,39] addressed the SPoF problem and provided solutions to increase the scalability and improve the availability; however, the rest of the papers did not address this problem and adopted different mechanisms to ensure other network requirements.

Some proposals, such as the work presented in [12,13,15,20,25], were facilitated by the SDN approach to improve the network requirements, but only the work presented in [25] presented a novel idea to solve the SPoF. In addition to SDN technology, in [15], the authors took NFV technology into account and tried to improve the end-to-end delay without considering the importance of the Fog Layer for IoT devices. Instead of Fog and SDN, in [17], the authors utilized the NFV and presented a limited solution for the SPoF problem as well as achieving network requirements other than security.

The work presented in [14,19,26] deployed the Fog Layer near the IoT domain; in this way, they solved the network management problems and increased the scalability. Some research contributions highlighted other aspects in current standards; for example, in [33], the node registration process is dealt with and mobility issues solved by introducing backbone boarder routers BBRs at the edge of the LLN. In contrast, in [29], the authors introduced a node address configuration method and provided a context (CO) dissemination scheme within 6LoWPAN. In [39], the authors closely addressed the same SPoF problem in the standard [3] that we are addressing regarding the synchronization of 6LoWPAN gateways to solve the SPoF and other services, but their solution is protocol-dependent, which limits its scalability.

### Why Neither SDN or “Classical” Approaches Can Be the Solution (Alone)

SDN and classical protocols have their respective limitations. As a matter of fact, the SDN controller, PAN coordinator, 6LBR and DoDAG Root are all SPoFs, and their operations are vital for a system’s resiliency. There are no standardized protocols for the North–South and East–West interfaces; moreover, the network becomes more unreliable and vulnerable when more than one functionality is running on a single node.

SDN is widely considered to be a possible method to improve network reliability. However, the whole reliability is based on the assumption that a reliable and secure channel is available between the SDN controller and the SDN switches. This assumption is valid for wired networks, where TCP and TLS can be used. In the IoT domain, this cannot be assumed; moreover, the SDN controller itself must be located in a high-security zone to prevent attacks on its availability.

All classical protocols in [2,3,4,5] are standardized, but they basically based on the *Tree* topology, where a single root is responsible for all the management services; for this reason, they suffer from multiple SPoF elements, leading to a global lack of resiliency.

We believe that both approaches can be used to ensure network resiliency, as they are effective on different network segments. However, when used in tandem, they can successfully ensure network resiliency.

## 5. Proposed Architecture

As discussed in previous Sections, the main issues in LLN reliability are related to devices whose functionality is essential for the network management (routing, address management, etc.). A targeted attack on one of these devices can jeopardize the network. Our proposal aims at mitigating this risk by moving the critical functionalities into a section of the network which is easier to manage and control and by providing redundancy and resiliency through virtualization [40]. In this way, we are also able to solve the SPoF problem and achieve the core requirements previously discussed. In this section, we present our architecture, properly integrating current standards, and some necessary discussion to strengthen the 6LoWPAN architecture.

Without loss of generality, we will focus on the 6LBR, RPL Root, and PAN coordinator functionalities. Traditionally, these three functions are implemented in the same device, which also acts as a gateway between the LLN and the Internet. Even though these functionalities might be split in different devices, this does not help to increase the resiliency of the network. In contrast, it creates a burden for the management, as multiple devices might be compromised, and the loss of even one of them will affect the whole system.

Our proposal relies on moving all the critical functionalities in the Fog domain, where they can be properly protected from attacks, and they can obtain more resilience thanks to the use of the NFV. Our architecture is based on three levels; i.e., the Fog domain, access network domain and LLN domain.

**Figure 4:**
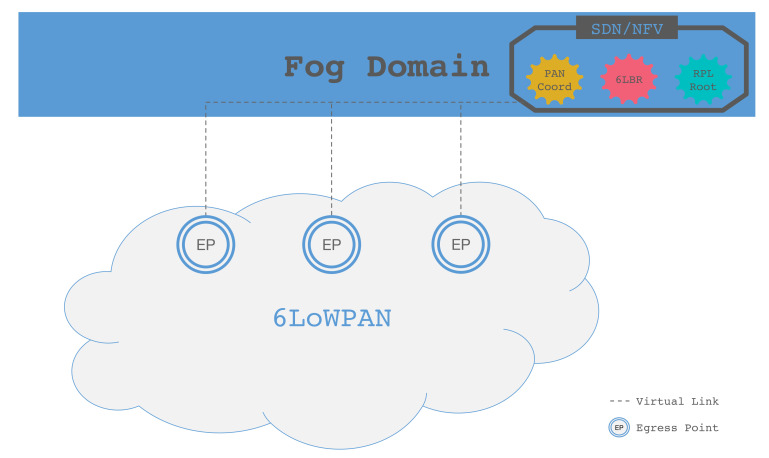
Fog-based LLN architecture.

As shown in Figure 4, EPs are installed at the edge of the LLN domain. Each EP is connected to the Fog domain through an access network, which can be assumed to have low latency and high reliability. Now, we will present the functionalities and requirements against each domain.

### 5.1. LLN Domain

The LLN Domain is composed of all the LLN devices, and we assume that it uses the latest IETF proposals for LLNs. This includes, but is not limited to, 6LoWPAN [9,10], 6LoWPAN-ND [3,4] and RPL [5]. These standards ensure proper resilience in the network, provided that the physical topology of the network allows redundant paths for the EPs.

To this end, it is important to ensure that the failure of a small portion of the devices leads to network partitioning. In other terms, the physical network topology should be as meshed as possible, eventually deploying nodes in which the network does not satisfy the necessary redundancy. To analyze this case, it is possible to apply graph theory; e.g., to ensure that there are no nodes that have a betweenness centrality [41] significantly different than the others. Under these assumptions, it is safe to consider the network as a whole as resilient, because even if a device is removed, the routing can quickly recover from the failure.

Inside the LLN, security and resiliency might also be improved by using MAC-level encryption, address shuffling [42] and other techniques to prevent attacks on single devices. However, we consider these aspects as beyond the scope of the current discussion.

### 5.2. Access Domain

The role of the this domain is to connect EPs to the Fog domain. Without loss of generality, we can assume that it can be considered as more reliable and secure by keeping in mind the nature of the LLN and its related standards. As an example, the access domain might rely on 5G, Ethernet or any system that allows the EPs to be connected to the Fog domain. The only requirement for the access network is to be able to configure a virtual point-to-point link between each EP and the Fog-based network functions. This can be accomplished though well-known network management procedures and can involve the use of SDN, encrypted tunnels, etc.

**Figure 5:**
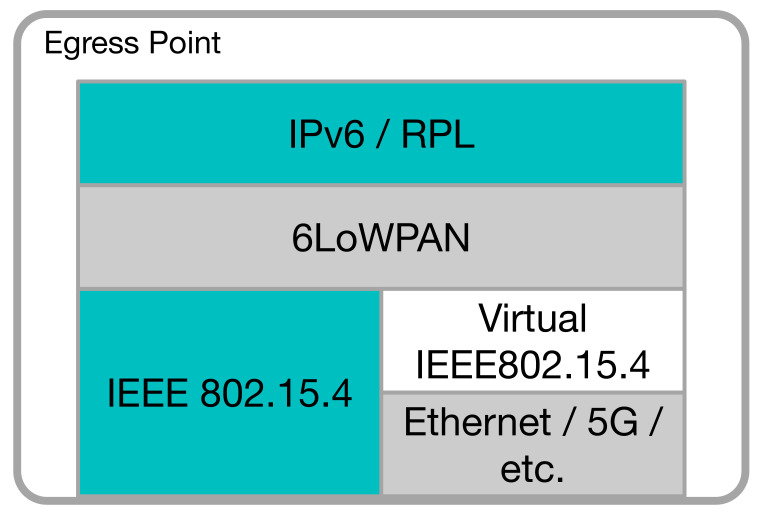
Egress Point architecture.

The EPs play a central role in the proposed architecture, which is shown in Figure 5. With respect to a normal LLN EP, where it acts as a gateway between the LLN and the Internet, in our architecture, the EP simply forwards the packets from the LLN to a set of devices implementing the necessary functionalities (i.e., PAN coordinator, 6LBR, RPL root, etc.) within the Fog domain. We refer to these in the following as LLN root functions, and they will be discussed in Section 5.3.

From an architectural point of view, EPs behave as the first hop set of nodes connected to the LLN root functions. To this end, it is necessary to have the following:

(1) A virtual 802.15.4 interface.
(2) A reliable and secure link between the LLN node and the LLN root functions.

The virtual 802.15.4 interface is necessary to enable seamless communication between the EPs and the devices implementing the LLN root functions. The link between the EP and the LLN root functions can be, for example, a direct Ethernet link or, in a more complex environment, an encrypted virtual link; i.e., a Virtual Private Network (VPN) tunnel.

The IEEE 802.15.4 standard-based frames, which originated from the virtual IEEE 802.15.4 interface, are tunneled through the above-mentioned link toward the LLN root functions, where they are handled as if they originated from a normal LLN device.

### 5.3. Fog Domain

This domain is built and managed according to the best practices for Fog systems and can be enhanced by using SDN and NFV technologies. In particular, SDN can help in providing flexible and resilient network management, link resilience and traffic engineering, while NFV allows us to replicate and dynamically allocate resources to the LLN root functions, implemented as NFV elements. As a result, we can safely assume that the LLN root functions implemented in the Fog are scalable and resilient. Moreover, thanks to the computational capacity of the Fog domain, we can assume that the LLN root functions are protected through proper security systems; i.e., firewalls and intrusion detection systems (IDSs).

The LLN root functions implemented in the Fog domain are described below.

#### 5.3.1. Virtual PAN Coordinator

The tasks of the PAN coordinator (i.e., devices association and disassociation, beacon generation, etc.) are implemented in the Fog and are practically identical to those of a “real” PAN coordinator. Its connection to the real LLN is ensured by the EPs, which behave as LLN nodes.

#### 5.3.2. Virtual 6LBR

The role of the 6LBR is to bridge the 6LoWPAN network (where IPv6 headers are compressed) and the IPv6 network. Toward this end, the virtual 6LBR behaves as a normal 6LBR thanks to the virtual IEEE 802.15.4 interface present in the EPs.

#### 5.3.3. Virtual RPL Root

Similar to the virtual 6LBR, the virtual RPL Root is practically identical to a traditional RPL root. The only difference is in how the metrics of the links between the root and the EPs are measured.

The virtual links between the RPL root and the EPs are different from the links present in the LLN. As a consequence, it is necessary to define how the metrics [43] of these links are collected. As an example, the “hop count” metric might be kept identical, while the “link reliability” metric might require a new definition.

**Figure 6:**
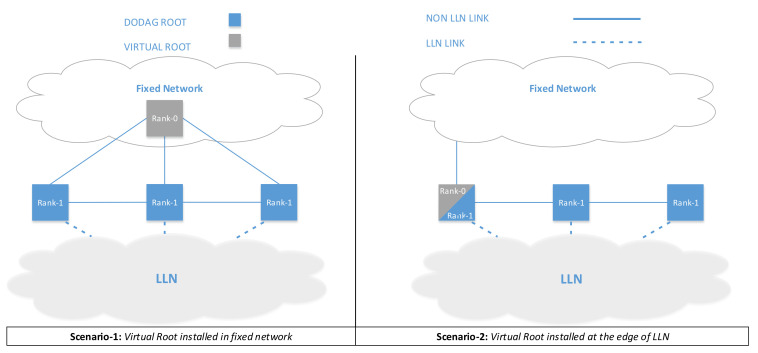
Virtual DoDAG Root placement scenarios.

To further enhance the routing resiliency, it is possible to implement the virtualization suggested RFC 6550 [5], where a virtual RPL root is implemented in the non-LLN section of the network and each gateway between the LLN and non-LLN domain is orchestrated by the Virtual RPL root. The standard proposes two scenarios, as shown in Figure 6. Our proposed architecture fully enables the first scenario foreseen in the standard, shown in the figure as Scenario 1. On the contrary, Scenario 2 (which is also foreseen by the standard) does not seem to enhance the network resiliency, as the RPL root (also acting as a virtual root) cannot be fully protected by the Fog domain.

#### 5.3.4. Virtual 6LoWPAN Backbone Router

A 6LoWPAN backbone router (6BBR) [6] enables seamless connectivity between an LLN and an IPv6 network, behaving as a routing registrar that provides proxy-ND services [4]. The use of a 6BBR allows us to avoid the use of a prefix for each LLN, and we believe that its use will be widespread in future LLN deployments. In the proposed architecture, the 6BBR can also be implemented as a network virtual function, further increasing the reliability and scalability of the “trenitalia” network.

### 5.4. Architecture Evaluation

**Figure 7:**
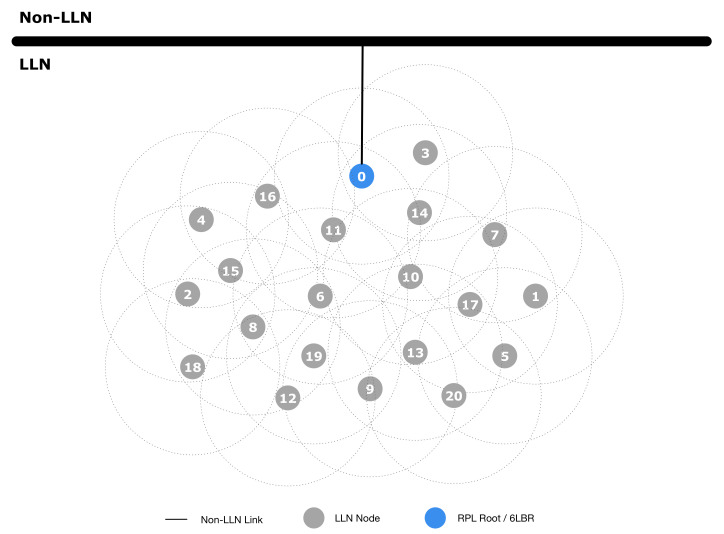
Conventional network setup.

**Figure 8:**
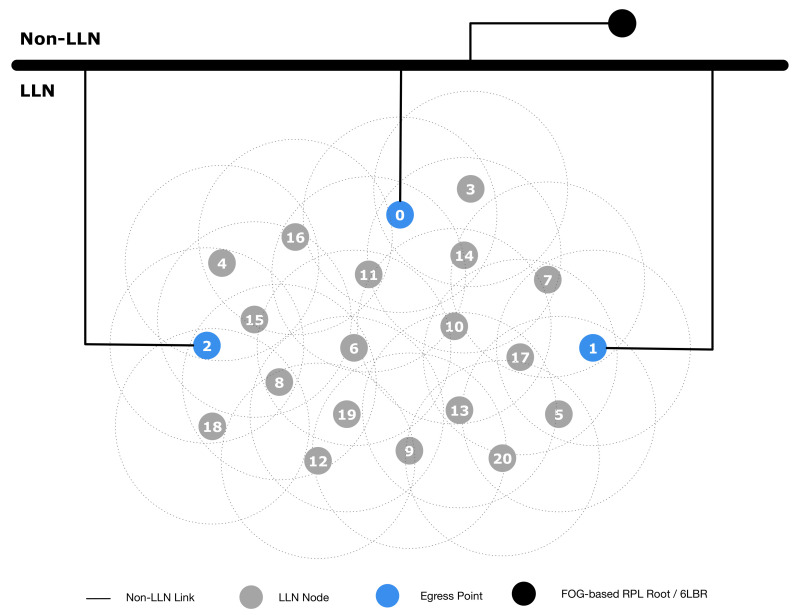
Proposed network setup.

To evaluate the effectiveness of our architecture, we performed some tests using the well-known ns-3 simulator (https://www.nsnam.org). In the simulation setup, we considered two scenarios: the first simulation scenario is shown in Figure 7, which is, as highlighted in Section 4, the classical conventional network layout; the second scenario represents our proposed architecture, and it is shown in Figure 8. The conventional network layout (Figure 7) is indeed composed of one gateway, acting as PAN coordinator, 6LoWPAN endpoint and RPL root, while the proposed network layout (Figure 8) includes multiple EPs and an NFV based node in the Fog domain performing the PAN coordinator, 6LoWPAN endpoint and RPL root functions. The simulation parameters to evaluate both scenarios are shown in Table 2.

In order to simulate an attack (or a device failure), we added some artificial noise to the signal received and sent by the device being attacked. This is equivalent to adding a jammer in the proximity of the attacked device or to physically tampering with the device antennas. The noise is powerful enough to prevent any signal being correctly sent or received by the device under attack.

In the first experiment, we used the conventional network setup as shown in Figure 7, where Node 0 (i.e., Gateway) is connected to the Internet. The attack is performed at Node 0 at second 15. After the attack, as shown in Table 3, all the sensor nodes become unreachable.

In the second experiment, we used the proposed network setup shown in Figure 8, where Nodes 0, 1 and 2 are EPs, and the NFV based node in the Fog domain is connected through reliable non-links. The results are presented in Table 4. In this experiment, we attacked the EPs (Node 2, 1 and 0), respectively, at seconds 15, 30, and 45. To better understand the network recovery time, we increased the RPL DoDAG version after the attack detection. This effectively detached all the nodes from the root and forced a new join procedure. As shown in the table, the network quickly recovers a fully functional state after each failure, and it becomes unavailable only when the last EP is successfully attacked.

The tests fully confirm the effectiveness of the proposed architecture. As a matter of fact, in the conventional network setup (Figure 7), an attack on the gateway node completely disables the whole LLN. Moreover, since the LLN nodes are disconnected, they could start to perform recovery mechanisms (i.e., find a new network), enabling the attacker to perform a secondary attack such as a gateway impersonation. On the contrary, in our proposed architecture, an attack on one (or more) EPs has limited or no effect on the network operations.

It is not necessary to remark that an attack on all the EPs is far more difficult to perform, and an adverse event (i.e., a failure) involving all the EPs at the same time is even less likely.

**Figure 9:**
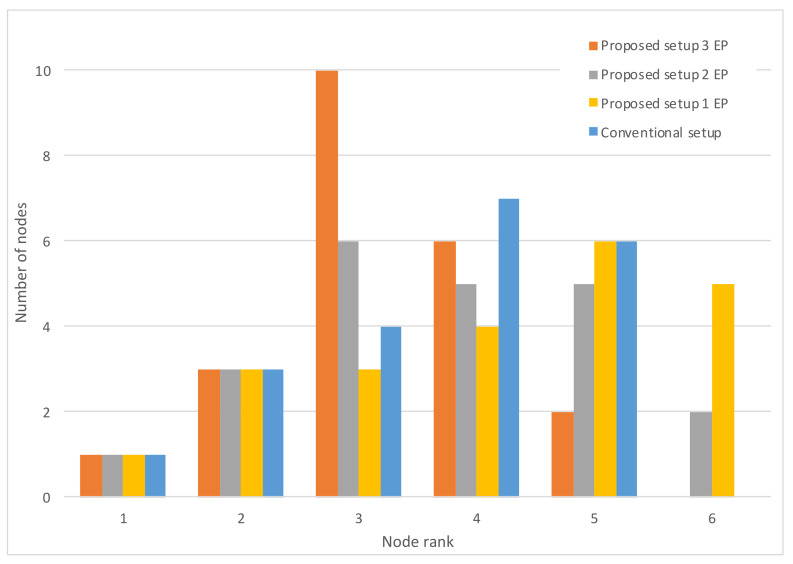
Histograms of node ranks.

Figure 9 shows the node rank histogram with a varying number of working EPs. It is evident that the introduction of the EP functionality has another benefit on the network, other than increased resiliency: the LLN maximum rank is smaller, with more nodes concentrated on ranks very close to those of the EPs (rank 2 nodes). This has many potential benefits for the network: less energy consumption, less latency, etc.

As a matter of fact, the possibility to use EPs goes beyond the pure network resilience, as they can become both an optimization parameter in the LLN (EP number and position), and a further security measure; e.g., by using “sleeping” EPs which are activated when an attack is detected.

One point that has not been studied in the present work, and that will be the subject of future research, is the network convergence time. RPL (which is the major driver for the network stability and convergence) is an extremely complex protocol, whose convergence is dependent on multiple parameters and which can be influenced by multiple factors (e.g., variable-period trickle timers, network failure detection methods, traffic patterns, network topology, objective functions, etc.). As a consequence, the study will have to involve multiple scenarios with varying numbers of nodes in different topologies (both synthetic and real).

## 6. Conclusions and Future Works

The utilization of IoT devices in our social life and ongoing research work is rapidly increasing, but this work also brings some important challenges. Some of them are required to fulfill the demands of the market, and they need to be addressed in way that strengthens IoT systems in the design process while developing it. All the related standards, IEEE 802.15.4 [2], 6LoWPAN-ND [3,4] and RPL [5], are based on the Tree topology, where the “root” node is responsible for all management services. Beside this, when a single node—i.e., a gateway—becomes a root node for an LLN, then it represent a single point of failure. Keeping in mind this problem, our proposed architecture provides significant scalability and availability by connecting multiple EPs to the Fog domain. The Fog domain also gives the opportunity to fulfill the network and security managements.

Our proposed architecture provides a way to deploy a resilient network to fulfill the core requirements and solution of decoupling of different services of root nodes. The simulation results fully confirm the validity of the approach. Moreover, the proposed architecture enables enhanced security countermeasures; e.g., by using “ghost” EPs, which are activated when an attack is detected.

Regarding future research, there are still open points that should to be studied to further improve the efficiency and resilience of IoT systems:

1. Virtual DoDAG root: The RPL standard [5] has not described the detailed functionality of the VDR. There is a need to define its proper functionalities so that it can harmonize all attached DoDAG roots and their designated LLNs.
2. Optimal RPL: The RPL protocol convergence time is high due to the inherent features of the distance vector and source routing (non-storing mode); in contrast, the single DoDAG root has the ability to manage thousands of constrained nodes. However, the theoretical limit on a DoDAG root has not been defined yet. Similarly, there is no limit on VDR to maintain DoDAG roots. Thus, there are imperative limits necessary on both vital devices to obtain the optimal performance of the RPL protocol.
3. Multiple backbone boarder routers (BBRs): Seamless integration and proper synchronization among 6BBRs is mandatory so that they can handle and provide services to their vicinity, such as mobility, but there is a need for a mechanism for recovery in the case of 6BBR failure.
4. Mobility: 6LoWPAN-ND [3] does not support mobility for inter-LoWPAN en route over networks. Although standards [4,6] support the mobility requirements for a device moving from one LLN to the next, proper network management is required among 6BBRs for the registration of lifetimes against acquired addresses.
5. Anycast address: Utilization of anycast addresses in 6LoWPAN-ND and RPL is challenging and requires the synchronization between DoDAG roots and 6BBRs.
6. Traffic management: The primary 6BBR was responsible for all services at a time and became a bottleneck for the entire 6LoWPAN, because secondary 6BBRs worked as a back-up support in case of primary 6BBR failure [6]. Thus, there is a need for a proper traffic management mechanism for all 6BBRs.

## Figures and Tables

**Table 1:** State-of-the-art contributions and limitations.

Paper	Year	Contribution	Av	Sc	Se	Ma	SPoF
[13]	2020	A central control that jointly manages end-to-end, both the wired segments and the Industrial IoT domain.	○	◐	○	◐	○
[14]	2020	An aggregator based RPL for an IoT-Fog based power distribution system with 6LoWPAN.	◐	●	○	◐	◐
[15]	2019	SD-NFV based architecture to reduce the end-to-end delay and strengthen energy depletion in motes.	○	○	○	◐	○
[16]	2019	Provide multi-gateway synchronization protocol ByzCast to increase data availability and improves fault and intrusion tolerance in WSNs.	◐	◐	●	◐	●
[17]	2018	NFV based Operating room Innovation Center (OPIC) to investigate time and non-time critical applications.	◐	◐	○	◐	◐
[18]	2018	Provide synchronization scheme for multiple gateways to increase network capacity and proposed a scheme to reduce the energy waste and TSCH enhancement.	◐	●	○	◐	◐
[19]	2018	Provide interoperability in home automation system for Fog computing applications based on MQTT and ZigBee-WiFi Sensor Nodes.	○	◐	○	◐	○
[20]	2018	A role based security controller architecture to strengthen the security of IoT.	○	○	●	◐	○
[21]	2018	An end-to-end indoor air quality monitoring (IAQM) system provide interoperability and backup support in case of connection failure IP or radio.	◐	◐	○	◐	○
[22]	2017	Provide synchronization algorithm for multiple gateways to increase the availability and reliability of critical applications.	◐	◐	○	◐	◐
[23]	2017	Cost effective scheme for the selection of gateways and adaptation mechanism is used to increase the system capacity to cope dynamic change.	◐	○	○	◐	○
[24]	2016	Provide energy-efficient services, fault tolerance, load balancing and resource management.	◐	◐	◐	●	○
[25]	2016	An hierarchical SDN approach provide security and handle communications between clusters by an SDN cluster head managed by an SDN controller.	◐	●	◐	●	◐
[26]	2016	Cloud-based security architecture for medical WSNs, where Access Control supports complex and dynamic security policies.	○	◐	◐	◐	○
[27]	2015	Overly architecture for WSN based fire monitoring system that relies on a constrained application protocol, where a single WSN is shared by multiple applications.	○	◐	○	◐	○
[28]	2015	Secured cross layer architecture for IoT to improve security management by Adaptive Interface Translation Table (AITT).	◐	○	●	◐	○
[29]	2015	A new scheme that provide address configuration and context management and their distribution in 6LoWPAN-based architecture.	◐	◐	○	◐	○
[30]	2015	Architecture facilitate interoperability, support low power operations, offers service discovery, registration and authentication mechanisms for IoT.	○	◐	◐	◐	○
[31]	2015	Provide a mechanism for fault tolerance at gateway and provide traffic management to avoid gateway being a bottleneck.	○	◐	○	◐	○
[32]	2015	A new design of gateway with the integration of 6LoWPAN adapter layer in a Network Adapter Driver (NAD) of computer.	○	○	○	◐	○
[12]	2015	A stateful approach to make programmable sensor nodes by reducing the amount of information exchanged between sensors and SDN controllers.	○	◐	○	◐	○
[33]	2015	Efficient-Neighbor Discovery that advertise reachability to a registered addresses and BBR solve the problem of node mobility.	○	◐	○	◐	○
[34]	2015	An automatic monitoring and tracking system for patients, biomedical devices within hospitals, that provide visibility of the motes and perform information management.	○	○	○	◐	○
[35]	2014	Analyzed different solutions for the integration of WSNs and Internet and provide Gateway solution for localization and tracking application.	○	○	○	◐	○
[36]	2014	A model for an Area Sensor Network (ASN) that connects heterogeneous networks and provide interoperability & scalability.	○	●	○	◐	○
[37]	2014	Provide dynamic and distributed load balancing scheme for multiple gateways to achieve global load fairness, network capacity, and reliability.	◐	◐	○	◐	◐
[38]	2014	Architecture for smart campuses and focused on data collection from sensors and its storage in the Cloud.	○	◐	○	◐	○
[39]	2014	Architecture based on multiple GWs and improve ND proxy, routing support, mobility and reliability for data delivery in 6LoWPANs.	●	◐	○	●	◐

Av = Availability, Sc = Scalability, Se = Security, Ma = Management, SPoF = Single Point of Failure; ○= no, ◐= partial, ●= yes.

**Table 2:** Simulation parameters.

Parameter Type	Value
Radio Range	About 100 m
802.15.4	Beaconless, always on
Propagation Model	Log-Distance
6LoWPAN Compression	IPHC - RFC 6282
RPL Constants	as per RFC 6550

**Table 3:** Conventional setup, with nodes ranked over time.

	Root										Sensor Nodes										
Time [s]	0	1	2	3	4	5	6	7	8	9	10	11	12	13	14	15	16	17	18	19	20
1	1	-	-	2	-	-	-	-	-	-	-	2	-	-	2	-	-	-	-	-	-
2	1	4	-	2	4	5	3	3	4	-	3	2	-	-	2	4	3	4	5	-	-
3	1	4	-	2	4	5	3	3	4	-	3	2	-	-	2	4	3	4	5	-	-
4	1	4	-	2	4	5	3	3	4	-	3	2	-	4	2	4	3	4	5	-	-
5	1	4	-	2	4	5	3	3	4	-	3	2	-	4	2	4	3	4	5	-	-
6	1	4	-	2	4	5	3	3	4	5	3	2	-	4	2	4	3	4	5	-	-
7	1	4	-	2	4	5	3	3	4	5	3	2	-	4	2	4	3	4	5	-	-
8	1	4	-	2	4	5	3	3	4	5	3	2	-	4	2	4	3	4	5	-	-
9	1	4	6	2	4	5	3	3	4	5	3	2	5	4	2	4	3	4	5	4	5
10	1	4	5	2	4	5	3	3	4	5	3	2	5	4	2	4	3	4	5	4	5
11	1	4	5	2	4	5	3	3	4	5	3	2	5	4	2	4	3	4	5	4	5
12	1	4	5	2	4	5	3	3	4	5	3	2	5	4	2	4	3	4	5	4	5
13	1	4	5	2	4	5	3	3	4	5	3	2	5	4	2	4	3	4	5	4	5
14	1	4	5	2	4	5	3	3	4	5	3	2	5	4	2	4	3	4	5	4	5
15	1	4	5	2	4	5	3	3	4	5	3	2	5	4	2	4	3	4	5	4	5
											**Root Node is Attacked**										
16	1	-	-	-	-	-	-	-	-	-	-	-	-	-	-	-	-	-	-	-	-
17	1	-	-	-	-	-	-	-	-	-	-	-	-	-	-	-	-	-	-	-	-
18	1	-	-	-	-	-	-	-	-	-	-	-	-	-	-	-	-	-	-	-	-

**Table 4:** Proposed network setup, with nodes ranked over time.

	Root										Sensor Nodes											
Time [s]	NFV	0	1	2	3	4	5	6	7	8	9	10	11	12	13	14	15	16	17	18	19	20
1	1	2	2	2	-	-	-	-	-	3	-	-	3	-	-	3	3	-	3	-	-	-
2	1	2	2	2	3	-	3	-	-	3	-	-	3	-	-	3	3	-	3	-	-	-
3	1	2	2	2	3	-	3	4	-	3	5	4	3	-	4	3	3	4	3	3	4	-
4	1	2	2	2	3	-	3	4	-	3	5	4	3	-	4	3	3	4	3	3	4	-
5	1	2	2	2	3	-	3	4	-	3	5	4	3	-	4	3	3	4	3	3	4	-
6	1	2	2	2	3	-	3	4	-	3	5	4	3	-	4	3	3	4	3	3	4	-
7	1	2	2	2	3	4	3	4	-	3	5	4	3	-	4	3	3	4	3	3	4	5
8	1	2	2	2	3	3	3	4	-	3	5	4	3	-	4	3	3	4	3	3	4	4
9	1	2	2	2	3	3	3	4	3	3	5	4	3	-	4	3	3	4	3	3	4	4
10	1	2	2	2	3	3	3	4	3	3	5	4	3	-	4	3	3	4	3	3	4	4
11	1	2	2	2	3	3	3	4	3	3	5	4	3	5	4	3	3	4	3	3	4	4
12	1	2	2	2	3	3	3	4	3	3	5	4	3	5	4	3	3	4	3	3	4	4
13	1	2	2	2	3	3	3	4	3	3	5	4	3	5	4	3	3	4	3	3	4	4
14	1	2	2	2	3	3	3	4	3	3	5	4	3	5	4	3	3	4	3	3	4	4
15	1	2	2	2	3	3	3	4	3	3	5	4	3	5	4	3	3	4	3	3	4	4
											**EP 2 is Attacked**											
16	1	2	2	2	3	-	-	-	-	-	-	4	-	-	-	-	-	-	3	-	-	-
17	1	2	2	2	3	-	-	-	-	-	5	4	-	-	4	-	-	-	3	-	6	-
18	1	2	2	2	3	-	-	-	-	-	5	4	3	-	4	-	-	-	3	-	6	-
19	1	2	2	2	3	5	3	-	3	-	5	4	3	-	4	-	5	4	3	-	6	-
20	1	2	2	2	3	5	3	-	3	-	5	4	3	6	4	-	5	4	3	-	6	-
21	1	2	2	2	3	5	3	-	3	-	5	4	3	6	4	-	5	4	3	-	6	-
22	1	2	2	2	3	5	3	-	3	6	5	4	3	6	4	4	5	4	3	7	6	4
23	1	2	2	2	3	5	3	4	3	5	5	4	3	6	4	4	5	4	3	6	5	4
24	1	2	2	2	3	5	3	4	3	5	5	4	3	6	4	3	5	4	3	6	5	4
25	1	2	2	2	3	5	3	4	3	5	5	4	3	6	4	3	5	4	3	6	5	4
26	1	2	2	2	3	5	3	4	3	5	5	4	3	6	4	3	5	4	3	6	5	4
27	1	2	2	2	3	5	3	4	3	5	5	4	3	6	4	3	5	4	3	6	5	4
28	1	2	2	2	3	5	3	4	3	5	5	4	3	6	4	3	5	4	3	6	5	4
29	1	2	2	2	3	5	3	4	3	5	5	4	3	6	4	3	5	4	3	6	5	4
30	1	2	2	2	3	5	3	4	3	5	5	4	3	6	4	3	5	4	3	6	5	4
											**EPs 1 and 2 are Attacked**											
31	1	2	2	2	-	-	-	-	-	-	-	-	3	-	-	3	-	-	-	-	-	-
32	1	2	2	2	3	-	-	-	-	-	-	4	3	-	-	3	-	-	5	-	-	-
33	1	2	2	2	3	-	-	4	-	-	-	4	3	-	-	3	-	-	5	-	-	-
34	1	2	2	2	3	-	6	4	4	-	6	4	3	-	5	3	-	-	5	-	5	-
35	1	2	2	2	3	-	6	4	4	-	6	4	3	-	5	3	-	-	5	-	5	-
36	1	2	2	2	3	-	6	4	4	-	6	4	3	-	5	3	-	-	5	-	5	-
37	1	2	2	2	3	5	6	4	4	-	6	4	3	-	5	3	-	4	5	-	5	-
38	1	2	2	2	3	5	6	4	4	6	6	4	3	6	5	3	5	4	5	7	5	6
39	1	2	2	2	3	5	6	4	4	6	6	4	3	6	5	3	5	4	5	7	5	6
40	1	2	2	2	3	5	6	4	4	5	6	4	3	6	5	3	5	4	5	6	5	6
41	1	2	2	2	3	5	6	4	4	5	6	4	3	6	5	3	5	4	5	6	5	6
42	1	2	2	2	3	5	6	4	4	5	6	4	3	6	5	3	5	4	5	6	5	6
43	1	2	2	2	3	5	6	4	4	5	6	4	3	6	5	3	5	4	5	6	5	6
44	1	2	2	2	3	5	6	4	4	5	6	4	3	6	5	3	5	4	5	6	5	6
45	1	2	2	2	3	5	6	4	4	5	6	4	3	6	5	3	5	4	5	6	5	6
											**EPs 0, 1, and 2 are Attacked**											
46	1	2	2	2	-	-	-	-	-	-	-	-	-	-	-	-	-	-	-	-	-	-
47	1	2	2	2	-	-	-	-	-	-	-	-	-	-	-	-	-	-	-	-	-	-
